# Active steering control based on preview theory for articulated heavy vehicles

**DOI:** 10.1371/journal.pone.0252098

**Published:** 2021-05-25

**Authors:** Jie Tian, Qingkang Zeng, Peng Wang, Xiaoqing Wang

**Affiliations:** 1 College of Automobile and Traffic Engineering, Nanjing Forestry University, Nanjing, China; 2 College of Mechanical and Electronic Engineering, Nanjing Forestry University, Nanjing, China; 3 School of Public Administration, Nanjing University of Finance and Economics, Nanjing, China; Tongii University, CHINA

## Abstract

This paper investigates the active steering control of the tractor and the trailer for the articulated heavy vehicle (AHV) to improve its high-speed lateral stability and low-speed path following. The four-degree-of-freedom (4-DOF) single track dynamic model of the AHV with a front-wheel steered trailer is established. Considering that the road information at the driver’s focus is the most clear and those away from the focus blurred, a new kind controller based on the fractional calculus, i.e., a focus preview controller is designed to provide the steering input for the tractor to make it travel along the desired path. In addition, the active steering controllers based on the linear quadratic regulator (LQR) and single-point preview controller respectively are also proposed for the trailer. However, the latter is designed on the basis of the articulation angle between the tractor and trailer, inspired by the idea of the driver’s single-point preview controller. Finally, the single lane change maneuver and 90^o^ turn maneuver are carried out. And the simulation results show that compared with the single-point preview controller, the new kind preview controller for the tractor can have good high speed maneuvering stability and low speed path tracking ability by adjusting the fractional order of the controller. On this basis, three different AHVs with the same tractor are simulated and the simulation results show that the AHV whose trailer adopts the single-point preview controller has better high-speed lateral stability and low-speed path tracking than the AHV whose trailer adopts the LQR controller.

## 1. Introduction

With the development of automotive computer-aided engineering (CAE) [1–4], automotive electronic control technology [5, 6] and intelligent vehicle technology [7], and the study on the influencing factors of traffic accidents [8], articulated heavy vehicles (AHV) also develop rapidly with the demand of efficient transportation of goods in recent years. Conventional AHVs are usually equipped with non-steerable trailers, which exhibit jack-knifing, trailer swinging, roll-over, trailer off-tracking and so on, and result in poor high-speed lateral stability and low-speed path following [9, 10].

The lateral stability and path following are usually evaluated by rearward amplification (RWA) and path-following off-tracking (PFOT), respectively. And the definition of the RWA is the ratio of the peak lateral acceleration of the trailer’s center of gravity (CG) to that of the tractor’s CG under an obstacle avoidance lane-change maneuver [11, 12]. The PFOT refers to the maximum radial offset between the path of the tractor’s front-axle and that of the trailer’s rear-axle [13, 14]. And a number of passive trailer steering systems have been developed, which can improve the low-speed performance at the cost of worse high-speed performance. However, the active steering of the tractor and the trailer can improve both the low and high-speed performances of the AHVs.

Several control strategies, i.e., active yaw torque controls at the truck CG, dolly CG and trailer CG were proposed for a six-axle truck/full-trailer, and the controller performance index parameters were determined based on the target value of RWA. And simulation results indicated that the dynamic performance and roll stability can be improved effectively when active yaw torque was applied to the truck or the last trailer [12]. For a tractor with three full-trailers, the command-steering of the trailers’ front axles were designed proportionately to the articulation angles between the tractor and corresponding trailers, and the optimal linear quadratic regulator (LQR) controller based on insights gain was also proposed. Simulation results demonstrated that the latter was effective for all speeds [13]. The front axle steering angle of the full-trailer was assumed to be proportional to that of the tractor and solved by setting the tractor’s sideslip angle as zero. The effect of an additional steering wheel in the full-trailer’s front axle on the directional response during lane change at high speed and 90^o^ turn maneuver at low speed was analyzed, which demonstrated that the trailer’s active steering can increase the directional stability and reduce the lateral force during steady turning [15].

Active steering control strategies were developed for the tractor/semi-trailer to achieve the accurate path following. Specifically, the low-speed controller based on kinematics and the high-speed controller based on dynamics were designed. In the intermediate speed range the two controllers were blended together using a speed-dependent gain. And the simulation results showed that the directional performance can be improved significantly by the active steering throughout the speed range [16]. In order to reduce the low-speed off-tracking of the tractor/semi-trailer whose all wheels were steerable, two-layer controllers composed of fuzzy controller and PID controller for both the tractor and the semi-trailer were independently designed to ensure that the end points of both the tractor and the trailer can exactly follow the path of the tractor’s first point. Simulation results confirmed the control effects [17]. For the directional control of a tractor/semi-trailer, the yaw rate, lateral velocity of the tractor, and the articulation angle were selected as the control variables, and a LQR was designed based on the linear model of the articulated vehicle to make the control variables follow the desired responses. And a nonlinear 14 DOF model was used to evaluate the proposed control method. Simulation results of the high-speed lane change maneuver on a slippery road showed that the directional behavior of the articulated vehicle was improved [18]. A linear vehicle model of tractor/semi-trailer was constructed. Based on the optimal control theory, the ‘virtual driver’ steering control for the semi-trailer was designed to minimize the path-tracking deviation of trailer rear end relative to the path of the hitch point, and improve its roll stability as well as. And the control effect was verified by the simulation results [19]. A comprehensive design framework for multi-semi-trailer AHV lateral stability performance optimization was proposed. The framework included active trailer steering, trailer differential braking, active roll control or coordinated control of the three. The proposed framework and performance indicators can effectively identify expected variables and reliably predict the performance range of AHV with active steering system [20].

The 5 degree-of-freedom (DOF) model of the tractor/semi-trailer was established. Based on the model predictive control, the active semi-trailer steering controller was designed to comprehensively control the path and attitude by making the trailer’s path and yaw angle follow the desired ones and as well as minimizing its sideslip angle. Simulation results indicated that the proposed controller can greatly improve its lateral stability and off-tracking [21]. A 3 DOF model of the tractor/semi-trailer with steered semi-trailer axles was built. The active steering controller based on LQR of the tractor and the trailer were proposed to make both the tractor and the trailer follow the desired yaw rate and minimize their side-slip angles at the same time. Simulation results showed that the active steering controller can improve maneuverability at low speed and lateral stability at high speed [22].

For a double combination (tractor-semitrailer-dolly-semitrailer), a robust static output feedback controller was designed to improve its high-speed lateral performance by the active steering of the dolly unit. Both H_∞_ and H_2_ methods were proposed to synthesize the controller’s robust against the cornering stiffness parameters of the tires in the semi-trailers. Simulation results verify the significant reduction in rearward amplification of yaw rate and high-speed transient off-tracking [23]. Considering the parametric uncertainty of the payload, a robust recursive regulator was proposed for the truck/semi-trailer to minimize the given cost function. Simulation results showed that the proposed method had better performance than H∞ controller in terms of robustness, lateral stability, driving smoothness and safety. And the main advantage of the proposed controller was that it did not depend on the offline adjustment of tuning parameters [24]. Four different controllers, i.e., LQR, sliding mode control (SMC), nonlinear sliding mode control (NSMC) and mu-synthesis (MS), were designed for active steering system of a tractor/semi-trailer in terms of the directional performance. And their robustness was examined by which the steering angles of the tractor rear and semitrailer axle wheels were manipulated. Simulation results indicated that the MS was the most robust to the uncertainties of the trailer mass and the longitudinal trailer CG position [25].

In conclusion, many active steering controllers including LQR, SMC and H_∞_, which mainly concentrated on either [15, 20, 23–25] or both [21, 22] of the low-speed path following and high-speed lateral stabilities, have been proposed for the AHVs, especially for the tractor/semi-trailer. However, only a few controllers can simultaneously ensure low-speed path tracking and high-speed lateral stabilities and the focus previewer controller was never used in these studies. In this paper a focus preview controller based on the fractional calculus for the tractor is proposed to provide the steering input for its front wheels so that the tractor can drive along the ideal path. And a single-point preview control is designed for the trailer to further improve the performance of the AHV, which is also never reported in the relative literatures.

In this study the contributions are as follows: (1) A focus preview controller based on the fractional calculus is introduced for the tractor of the AHV due to the characteristics of the driver’s sight. (2) The active steering controllers for the trailer based on the LQR and the single-point preview are designed. And the latter is designed on the basis of the articulation angle between the tractor and trailer, inspired by the idea of the driver’s single-point preview. (3) The simulations of the high-speed single lane change and low-speed 90^o^ turn maneuvers are carried out to obtain the optimal fractional orders of the focus preview controllers for the tractors, and verify the designed controllers for the trailer.

The rest of this paper is as follows: The 4 DOF single track dynamic model of the AHV with a front-wheel steerable trailer is established in Section 2. The active steering control strategy of the AHV is illustrated in Section 3. Section 4 introduces the fractional calculus to describe the driver’s sight characteristics, and designs the focus preview controller for the tractor. The LQR controller and single-point controller for the trailer are designed in section 5. Simulation results are analyzed in Section 6. Section 7 is the conclusion.

## 2. AHV model

**Fig 1:**
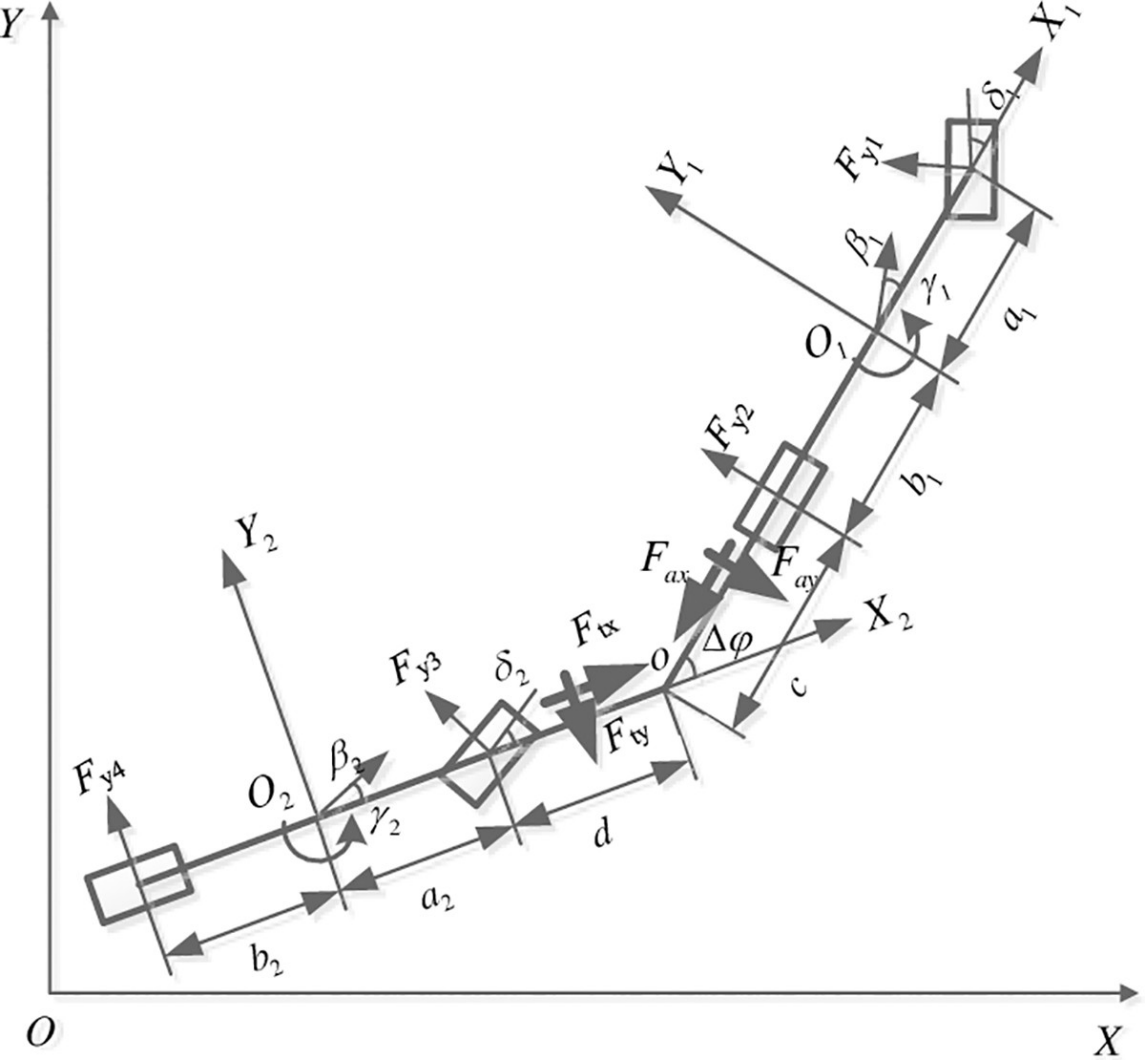
Dynamics model of AHV.

There are many types of AHV. However, the AHV studied in this paper is composed of a tractor and a full trailer, which connects with each other by a hitch at point *o*, and is presented as a single track vehicle in Fig 1.

As shown in Fig 1, the geodetic coordinate system is *XOY*, and the vehicle coordinate systems fixed at the CG of the tractor and trailer are *X*_1_*O*_1_*Y*_1_ and *X*_2_*O*_2_*Y*_2_, respectively. Ignoring the pitch, bounce motions and aerodynamic forces, and assuming the front wheels of the trailer can also steer, the motion of the tractor can be expressed as

$$\left\{ \begin{array}{l} \sum F_{Y1}=F_{y1}\cos\delta_{1}+F_{y2}+F_{ay}=m_{1}u_{1}\left(\gamma_{1}+{\dot{\beta }}_{1}\right)\\ \sum M_{z1}=F_{y1}a_{1}\cos\delta_{1}-F_{y2}b_{1}-F_{ay}(b_{1}+c)=I_{z1}{\dot{\gamma }}_{1} \end{array}\right.,$$ (1)

The motion of the trailer can be described as

$$\left\{ \begin{array}{l} \sum F_{Y2}=F_{y3}\cos\delta_{2}+F_{y4}-F_{ty}=m_{2}u_{2}\left(\gamma_{2}+{\dot{\beta }}_{2}\right)\\ \sum M_{z2}=F_{y3}a_{2}\cos\delta_{2}-F_{y4}b_{2}-F_{ty}(a_{2}+d)=I_{z2}{\dot{\gamma }}_{2} \end{array}\right.,$$ (2)

where *m*_*i*_ (*i =* 1,2) is the mass of the tractor/trailer; *I*_*zi*_ is the moment of inertia about *Z*_i_ axis of the tractor/trailer; *a*_*i*_ and *b*_*i*_ are the distances from the CG to the front and the rear axle of the tractor/trailer, respectively; *c* and *d* are the distances from the hitch point to the rear axle of the tractor and front axle of the trailer, respectively; *u*_*i*_ is the longitudinal velocity of the tractor/trailer, *β*_*i*_ and *γ*_*i*_ are the sideslip angle and yaw rate of the tractor/trailer, respectively; *δ*_*i*_ is the front-wheel steering angle of the tractor/trailer, Δ*φ* is the articulation angle between the tractor and trailer, *F*_*yj*_ is the lateral forces on the front and the rear tires of the tractor and trailer (*j* = 1,2,3,4), *F*_*ax*_ and *F*_*ay*_ are the longitudinal and lateral force acting on the tractor by the trailer at the hitch point, respectively; *F*_*tx*_ and *F*_*ty*_ are the longitudinal and lateral force acting on the trailer by the tractor at the hitch point, respectively.

Because the dynamic coordinates of the tractor and trailer are different, the following relations can be obtained by the velocity projection law from Fig 1.

$$\left\{ \begin{array}{l} u_{2}\cos\beta_{2}=u_{1}\cos\beta_{1}\cos\Delta \varphi +\left[\gamma_{1}(b_{1}+c)-u_{1}\sin\beta_{1}\right]\sin\Delta \varphi \\ \gamma_{2}(a_{2}+d)+u_{2}\sin\beta_{2}=u_{1}\cos\beta_{1}\sin\Delta \varphi -\left[\gamma_{1}(b_{1}+c)-u_{1}\sin\beta_{1}\right]\cos\Delta \varphi \end{array}\right.,$$ (3)

Assuming Δ*φ* and *β*_*i*_ are very small, then *u*_1_ = *u*_2_, *F*_*ay*_ = *F*_*ty*_, *F*_*ax*_ = *F*_*tx*_, and the following expression can be obtained from (2).

$$\beta_{2}=\beta_{1}+\Delta \varphi -\gamma_{1}\frac{b_{1}+c}{u}-\gamma_{2}\frac{a_{2}+d}{u},$$ (4)

Assuming that the vehicle lateral acceleration is less than 0.4g, the relationship between *F*_*yj*_ and *α*_*j*_ can be expressed as

$$F_{yj}=k_{j}\alpha_{j}\;j=1,2,3,4,$$

$$\alpha_{1}=\delta_{1}-\beta_{1}-\frac{a_{1}\gamma_{1}}{u_{1}},\;\alpha_{2}=\frac{b_{1}\gamma_{1}}{u_{1}}-\beta_{1},\;\alpha_{3}=\delta_{3}-\beta_{2}-\frac{a_{2}\gamma_{2}}{u_{2}},\;\alpha_{4}=\frac{b_{2}\gamma_{2}}{u_{2}}-\beta_{2},$$ (5)

where *k*_*j*_ and *α*_*j*_ are the cornering stiffness and sideslip angle of the front and the rear tires of the tractor/trailer.

In addition,

$$\Delta \dot{\varphi }=\gamma_{1}-\gamma_{2},$$ (6)

Submit the Eqs (3) and (4) into (1) and (2), and set *X* = [*β*_1_ *γ*_1_ *γ*_2_ Δ*φ*]^*T*^, the corresponding state Eqs of (1), (2) and (5) is as follows:

$$\dot{X}=AX+B_{1}\delta_{1}+B_{3}\delta_{3},$$ (7)

where *A* = −*M*^−1^*N*, *B*_1_ = *M*^−1^*H*_1_, *B*_3_ = *M*^−1^*H*_3_, *b*_11_ = *k*_i_(*a*_1_+*b*_i_+*c*)+*k*_2_*c*, *b*_21_ = *k*_1_+*k*_2_+*k*_3_+*k*_4_, $b_{12}=k_{1}(a_{1}+b_{1}+c)\frac{a_{1}}{u}-k_{2}\frac{b_{1}}{u}c+m_{1}u(b_{1}+c)$, $b_{22}=k_{1}\frac{a_{1}}{u}-k_{2}\frac{b_{1}}{u}-k_{3}\frac{b_{1}+c}{u}-k_{4}\frac{b_{1}+c}{u}+m_{1}u$, $b_{23}=-k_{3}\frac{d}{u}-k_{4}\frac{a_{2}+b_{2}+d}{u}+m_{2}u$, *b*_24_ = *k*_3_+*k*_4_, *b*_31_ = (*k*_i_+*k*_2_)(*a*_2_+*d*)+*k*_3_*a*_2_−*k*_4_*b*_2_, *b*_34_ = *k*_3_*a*_2_−*k*_*4*_*b*_2_, $b_{32}=\frac{(a_{2}+d)\left(k_{1}a_{1}-k_{2}b_{1}\right)+\left(b_{1}+c\right)\left(-k_{3}a_{2}+k_{4}b_{2}\right)}{u}+m_{1}u(a_{2}+d)$, $b_{33}=\frac{-k_{3}a_{2}d+k_{4}b_{2}\left(a_{2}+b_{2}+d\right)}{u}$,

$$M=\left[\begin{array}{cccc} m_{1}u\left(b_{1}+c\right) & I_{z1} & 0 & 0\\ \left(m_{1}+m_{2}\right)u & -m_{2}\left(b_{1}+c\right) & -m_{2}\left(a_{2}+d\right) & m_{2}u\\ m_{1}u\left(a_{2}+d\right) & 0 & I_{z2} & 0\\ 0 & 0 & 0 & 1 \end{array}\right],N=\left[\begin{array}{cccc} b_{11} & b_{12} & 0 & 0\\ b_{21} & b_{22} & b_{23} & b_{24}\\ b_{31} & b_{32} & b_{33} & b_{34}\\ 0 & -1 & 1 & 0 \end{array}\right],H_{1}=\left[\begin{array}{c} b_{11}-k_{2}c\\ k_{1}\\ k_{1}(a_{2}+d)\\ 0 \end{array}\right],H_{3}=\left[\begin{array}{c} 0\\ k_{3}a_{2}\\ k_{3}\\ 0 \end{array}\right].$$

## 3. Problem formulation

**Fig 2:**
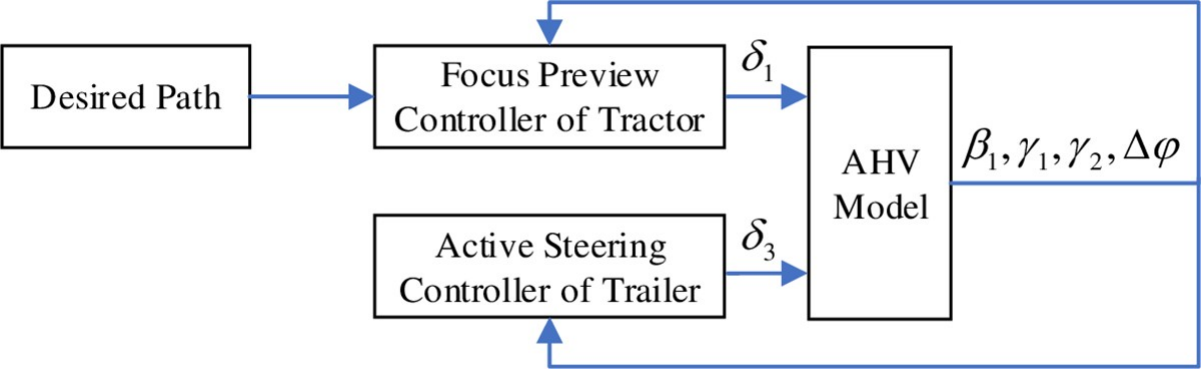
Control block diagram of AHV.

The AHV consists of a tractor and a full trailer, and the tractor does not bear the load of the trailer, but only provides the traction by the hitch to make the trailer move. That is to say, for the normal AHVs the trailer is only passively pulled by the tractor, which results in its poor path following performance when driving in a curve. Therefore, compared with a single vehicle the control of the AHV is relatively complex, which includes both of the controls of the front-wheel steering angles of the tractor and the trailer. And the proposed control block diagram is shown in Fig 2.

According to the desired path in the field of vision and the output signal of the AHV, the focus preview controller of the tractor will provide the front-wheel steering angle of the tractor, *δ*_1_, to the AHV model by calculating the lateral preview error, *y*_*d*_, and the deviation between the desired path and the actual path, *y*_*ε*_. In addition, the front-wheel steering angle of the trailer, *δ*_3_, can be obtained by the active steering controller of the trailer. Therefore under the joint action of *δ*_1_ and *δ*_3_, the AHV can drive along the desired path and have good performance.

## 4. Active steering control of tractor

In the normal driving behavior, the driver observes the external road information visually and transmits the information to the brain. The brain determines the preview distance according to the previous driving experience, and adjusts the steering wheel angle according to the deviation between the actual path and the ideal path of the vehicle, then realizes the change of the actual path through the change of the front wheel steering angle.

In the classic single-point preview model, it is usually assumed that the driver’s eyes only focus on the road information of a certain point ahead, while in the multi-point preview model, it is assumed that the driver focuses on the road conditions of a region ahead [26]. In fact, the drivers often not only focus on the road information of a certain point in front of them, but also take into account other road information in the field of vision. When we define the certain point, which the drivers pay attention to, as the focus of vision, the road information at the focus is the most clear, and the road information away from the focus is gradually blurred. This clarity and fuzziness of the vision can be reflected by the weight distribution, that is to say, the weight at the focus is the largest, and the weight away from the focus is smaller and smaller. In order to describe this sight characteristic, the fractional calculus is introduced, and the preview model established in turn is called focus preview model.

### 4.1. Fractional calculus

If the function of an independent variable, *x*, is *f*(*x*), the *α* order fractional calculus of *f*(*x*) can be recorded as follows,

$${}_{a}D_{x}{}^{\alpha }f(x)=f^{\alpha }(x)\;x\in \left(a,b\right),$$ (8)

where the value of *α* can be any complex number, *R*(*α*) is the real part. If *α*> 0, _*a*_*D*_*x*_^*α*^ is the fractional order derivation. Otherwise, it is the fractional order integration. When the natural number, *n*, is taken as the order *α* of the fractional calculus, then _*a*_*D*_*x*_^*α*^*f*(*x*) =*f*^*n*^(*x*), i.e., it becomes the integral derivative of *f*(*x*) in the general sense. When *α* = -*n*, it represents the integer order integral.

There are many definitions of fractional calculus [27], here we adopt the Grünwald-Letnikov (G-L) definition as the follows.

$${}_{a}D_{x}{}^{\alpha }f\left(x\right)=\frac{1}{h^{\alpha }}\sum_{k=0}^{\left[\left(b-a\right)/h\right]}\omega_{k}^{\left(\alpha \right)}f\left(x-kh\right)\;k=1.2,\ldots .n,$$ (9)

where *h* is the space step, $\omega_{k}^{\left(\alpha \right)}$ is the normalized weight [19] and

$$\omega_{k}^{\left(\alpha \right)}={\left(-1\right)}^{k}\left(\begin{array}{l} \alpha \\ k \end{array}\right)={\left(-1\right)}^{k}\frac{\alpha !}{k!\left(\alpha -k\right)!},$$ (10)

which can be directly obtained by the following recursive formula.

$$\left\{ \begin{array}{ll} \omega_{0}^{\left(\alpha \right)}=1\\ \omega_{k}^{\left(\alpha \right)}=\left(1-\frac{\alpha +1}{k}\right)\omega_{k-1}^{\left(\alpha \right)}, & k=1.2,\ldots .n. \end{array}\right.,$$ (11)

It can be seen from (11) that when *α* changes between -1 and 0, there exist the inequality, $\omega_{k}^{\left(\alpha \right)}<\omega_{k-1}^{\left(\alpha \right)}<\omega_{0}^{\left(\alpha \right)}$, *k* = 1.2,….*n*. That is to say, the weight coefficient $\omega_{k}^{\left(\alpha \right)}$ becomes smaller and smaller with the increase of *k* when *α* is fixed. And the different *α* will also result in different weight coefficients.

**Fig 3:**
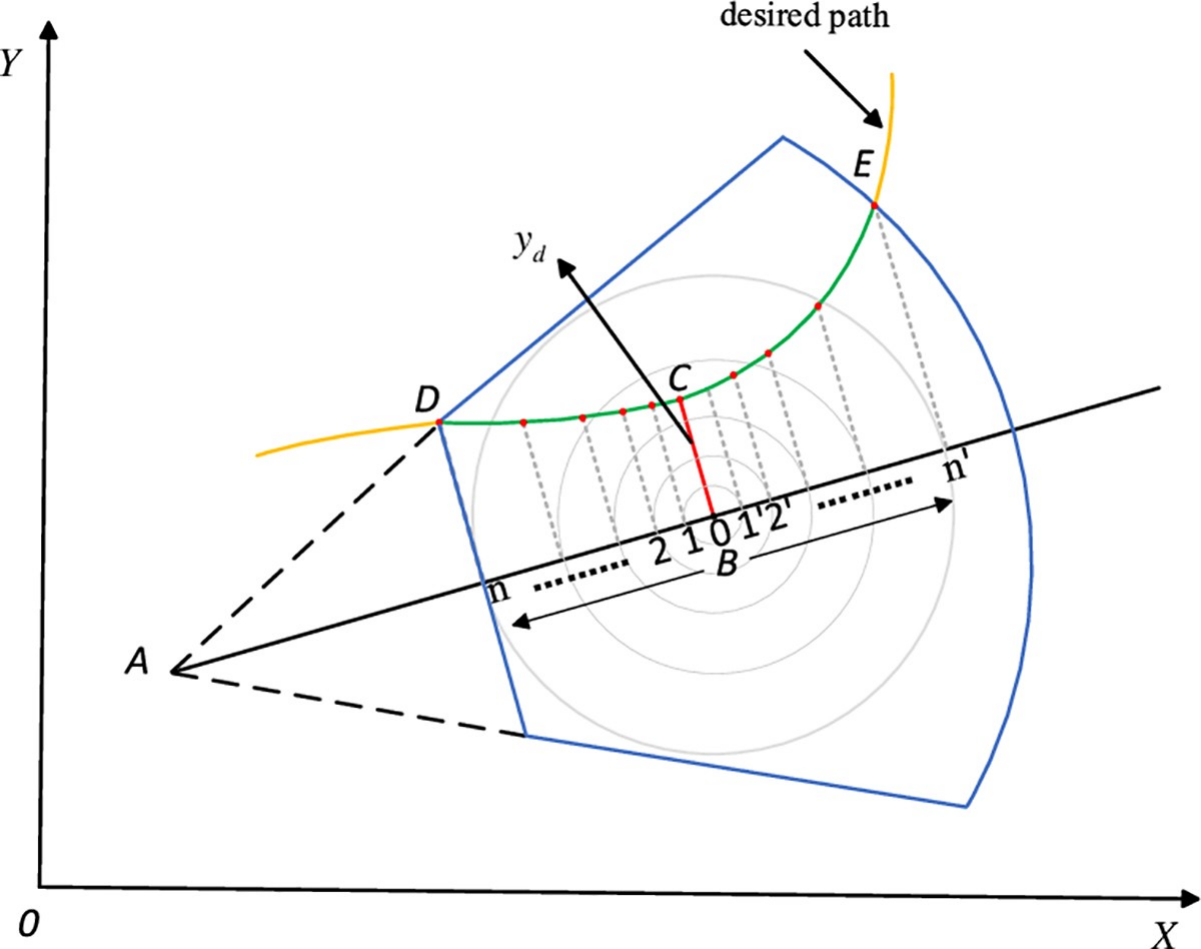
Sight characteristics of driver.

Specifically, we can establish the driver’s field of vision as shown in Fig 3, where the driver’s field of vision is represented by the dark blue sector area, point *A* is the driver’s position, point *B* is the driver’s focus located ahead in the longitudinal axis direction of the tractor. The distance between *A* and *B* is the preview distance, *L*. *D* and *E* are the closest and farthest points on the ideal path in the field of vision. The intersection point of the line perpendicular to *AB* through *B* and the ideal path is C, and *BC* is the distance between the focus and ideal path, *y*_*d*_, *θ* is the heading angle of the tractor. The desired path is represented by *f*(*x*). And at the point *B*, *k = 0* and the weight coefficient is the biggest. With the point away from *B*, *k* becomes larger and the weight coefficient becomes smaller. Therefore, we can make good use of the weight characteristics of the fractional calculus to simulate the driver’s sight characteristic. And this is the reason why we introduce the fractional calculus.

### 4.2. Design of focus preview controller

**Fig 4:**
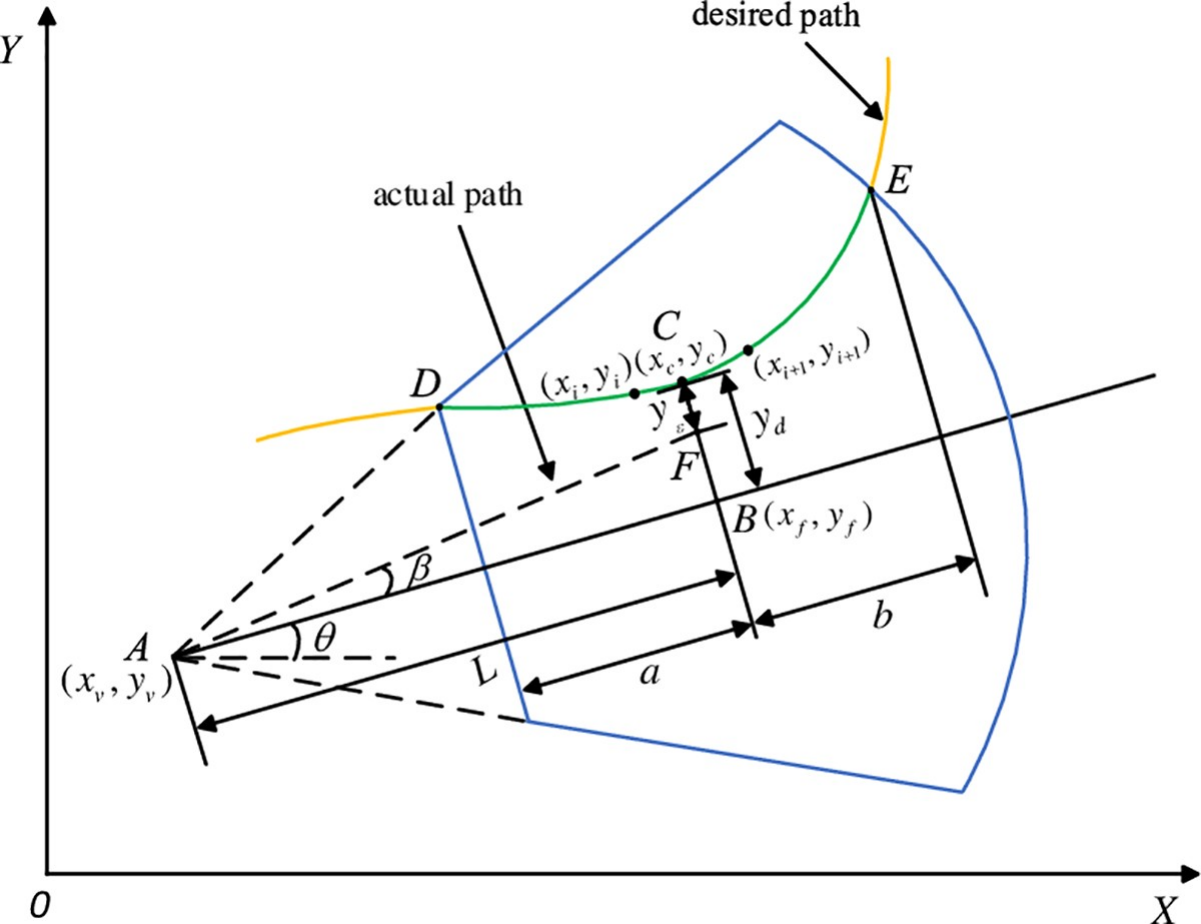
Schematic of focus preview.

The purpose of the focus preview controller is to simulate the driver’s driving behavior and obtain the front wheel steering angle of the tractor so that the AHV can travel along the ideal path. The focus preview diagram is shown in Fig 4.

Because both of the road information of the focus and others in the visual field should be pay attention by the driver, and the road information at the focus is the most clear while those away from the focus are gradually blurred, the fractional calculus is introduced to comprehensively consider the path information within the field of view. And the distance between *B* and *C* can be expressed as:

$$y_{d}=\frac{{}_{x_{D}}D_{x_{B}}^{\alpha }f(x){+}_{x_{B}}D_{x_{E}}^{\alpha^{\prime }}f\left(x^{\prime }\right)}{x_{E}-x_{D}},$$ (12)

Assuming that the tractor is running in a straight line with the current heading angle, the deviation between the ideal path and the actual path can be expressed as

$$y_{\varepsilon }=y_{d}-L\beta_{1},$$ (13)

Based on the deviation, *y*_*ε*_, the driver will adjust the steering wheel to get the front wheel steering angle of the tractor, *δ*_1_, which can be expressed as follows.

$$\delta_{1}\left(t\right)=K_{m1}y_{\varepsilon }\left(t-T_{s1}\right),$$ (14)

where *K*_*m*1_ is the system gain, *T*_*s*1_ is the delay time of the steering system.

Its Laplace transformation can be obtained as

$$\delta_{1}\left(s\right)=\frac{K_{m1}y_{\varepsilon }\left(s\right)}{e^{T_{s1}s}},$$ (15)

Considering *T*_*s*1_ is very small, the higher-order terms of $e^{T_{s1}s}$ are close to zero, keep the first two terms and carry out the Laplace inverse transformation, we can have the differential equation of the front wheel steering angle of the tractor as follows, which is the control strategy of the focus preview controller.

$${\dot{\delta }}_{1}\left(t\right)=-\frac{1}{T_{s1}}\delta_{1}\left(t\right)+\frac{1}{T_{s1}}K_{m1}y_{\varepsilon }\left(t\right),$$ (16)

### 4.3. Determination of preview point and direction of front-wheel steering angle

Seen from (16), it is necessary to calculate the driver’s preview deviation and sign at each discrete time in the process of numerical simulation of the AHVs, and take them as the decisive factors of the tractor’s front-wheel steering angle.

As shown in Fig 4, the coordinate of *A* and *B* are (*x*_*v*_, *y*_*v*_), and (*x*_*f*_, *y*_*f*_), respectively, the heading angle is *θ*, then

$$\{\begin{array}{c} x_{f}=x_{v}+L\cos\theta \\ y_{f}=y_{v}+L\sin\theta \end{array},$$ (17)

The straight line passing through *B* and perpendicular to the line *AB* can be described as,

$$\left(y_{f}-y_{v}\right)\left(y-y_{f}\right)+\left(x_{f}-x_{v}\right)\left(x-x_{f}\right)=0,$$ (18)

which intersects with the preview path at point *C* with coordinates (*x*_*c*_, *y*_*c*_), and *C* is the preview point.

Substitute (17) into (18), then

$$x\cos\theta +y\sin\theta =x_{f}\cos\theta +y_{f}\sin\theta .$$ (19)

Suppose the coordinates of the two points on the target preview route are (*x*_*i*_, *y*_*i*_) and (*x*_*i*+1_, *y*_*i*+1_), the line equation composed by them is

$$\left(x_{i+1}-x_{i}\right)\left(y-y_{i}\right)-\left(y_{i+1}-y_{i}\right)\left(x-x_{i}\right)=0,$$ (20)

The coordinate of *C*, (*x*_*c*_, *y*_*c*_), can be obtained by combining (19) and (20). If it satisfies the condition, *x*_*i*_≤*x*_c_≤*x*_*i*+1_, the coordinate (*x*_*c*_, *y*_*c*_) is the preview point *C*. Otherwise, *i* will be increased by 1, and continue to search until the calculation result meet the condition. After obtaining the preview point, the distance *y*_*d*_ between point *C* and point *B* is the preview deviation.

On this basis, it is necessary to further judge the direction of the front-wheel steering angle of the tractor. Here, point (*x*_*i*_, *y*_*i*_) and point (*x*_*i*+1_, *y*_*i*+1_) constitute vector *a*, point (*x*_*i*_, *y*_*i*_) and point *B* constitute vector *b*, then we can judge the preview point located on the left or right sides in front of the vehicle through the sign of the vector product of *a* and *b*.

## 5. Active steering control of trailer

Although the trajectory tracking of the tractor to the ideal path can be achieved through the above controller, the trailer’s following performance is certainly not ideal if it only follows passively without the active steering. Here two control strategies, the LQR control and single-point preview control, are proposed. And the former is used to compare with the latter.

### 5.1. Design of LQR controller

The LQR controller is an optimal dynamic controller designed based on the state space technology. The system model is a linear system in the form of state space, and its objective function is a quadratic function of state and control input. The quadratic problem is to select the control input to minimize the quadratic objective function under the constraints of the linear system.

The sideslip angle is the angle between the vehicle longitudinal and the motion direction of the vehicle CG, i.e., the tangential angle of the vehicle rotary circular showing the vehicle attitude in the uniform circular motion. When both of *β*_1_ and *β*_2_ are approaching to zero, the instant direction of the vehicle CG is close to the tangent direction of the rotating circle during the turning. And the smaller the value of the longitudinal angle between the tractor and trailer, Δ*φ*, the tractor and the trailer is more similar to drive in a line. Therefore, the performance index is constructed as follows and subjected to (7).

$$J=\int_{0}^{\infty }\left[q_{1}\left(\beta_{1}^{2}+\beta_{2}^{2}+\Delta \varphi^{2}\right)+q_{2}\delta_{3}^{2}\right]dt,$$ (21)

where *q*_1_ and *q*_2_ are the weighting factors that impose penalties upon the magnitude and duration of *β*_1_, *β*_2_ and Δ*φ*, and the active steering angle, *δ*_2_, respectively.

Note that the item on the right side of (21) represents the energy consumption of the system. Substituting (4) into (21), then (21) can be converted to a two-time matrix like this:

$$J=\int_{0}^{\infty }(X^{T}QX+U^{T}RU)dt,$$ (22)

where *U* = *δ*_3_, *R* = [*q*_2_],

$$Q=q_{1}\left[\begin{array}{cccc} 2 & -\frac{b_{1}+c}{u} & -\frac{a_{2}+d}{u} & 1\\ -\frac{b_{1}+c}{u} & {\left(\frac{b_{1}+c}{u}\right)}^{2} & \frac{\left(b_{1}+c)(a_{2}+d\right)}{u^{2}} & -\frac{b_{1}+c}{u}\\ -\frac{a_{2}+d}{u} & \frac{\left(b_{1}+c)(a_{2}+d\right)}{u^{2}} & {\left(\frac{a_{2}+d}{u}\right)}^{2} & -\frac{\left(a_{2}+d\right)}{u}\\ 1 & -\frac{b_{1}+c}{u} & -\frac{\left(a_{2}+d\right)}{u} & 2 \end{array}\right].$$

By solving the algebraic Riccati equation, the solution is the control vector of the form

U=−KX, (23)

where *K* is the control gain matrix with a dimension of 1×4.

### 5.2. Design of singer-point preview controller

In this section, the steering control strategy is given by referring to the driver’s single-point theory, so as to maintain the articulation angle between the tractor and the trailer in a stable range, reduce the lateral force interference between the trailer and the tractor, and improve the following performance and driving stability of the trailer.

As we all know, the smaller the value of the articulation angle between the tractor and the trailer, Δ*φ*, the trailer’s following performance is more better because the tractor and the trailer is more similar to drive in a line. Considering the driver’s preview control is that the driver constantly adjusts the steering wheel angle according to the deviation between the actual path and ideal path to make the vehicle travel along the idea path, we put forward the front-wheel steering angle control strategy of the trailer based on the single-point preview by analogy. That is to say, the front-wheel steering angle of the trailer is adjusted according to Δ*φ* to make the trailer run in a straight line of the tractor as much as possible, which can be expressed as follows.

$$\delta_{3}\left(t\right)=K_{m2}\Delta \varphi \left(t-T_{s2}\right),$$ (24)

where *K*_*m*2_ is the system gain, *T*_*s*2_ is the delay time of the steering system.

The Laplace transformation of (24) is as follows.

$$\delta_{3}\left(s\right)=\frac{K_{m2}\Delta \varphi \left(s\right)}{e^{T_{s2}s}},$$ (25)

Considering *T*_*s2*_ is very small, the higher-order terms of Taylor series expression of $e^{T_{s}s}$ are close to zero, retain the first two terms, the following control strategy can be obtained after Laplace inverse transformation.

$$\dot{\delta_{3}}=-\frac{1}{T_{s2}}\delta_{3}+\frac{1}{T_{s2}}K_{m2}\Delta \varphi ,$$ (26)

where *K*_*m*2_ is the system gain, *T*_*s*2_ is the delay time of the steering system.

## 6. Simulation results and analysis

For the AHVs, the single lane change maneuver and 90^o^ turn maneuver are important methods to examine the maneuvering stability at high speed and the path tracking performance at low speed. The parameters used in the simulation are as follows: *m*_1_ = 27715kg, *I*_*z*1_ = 19665kgm^2^, *a*_1_ = 2.0m, *b*_1_ = 3.135m, *m*_2_ = 29927kg, *I*_*z*2_ = 180117kgm^2^, *a*_2_ = 2.6m, *b*_2_ = 2.535m, *c* = 1.15m, *d* = 2.12m, *k*_1_ = 920000N/rad, *k*_2_ = 590000N/rad, *k*_3_ = *k*_4_ = 1200000N/rad.

### 6.1. Single lane change maneuver

**Fig 5:**
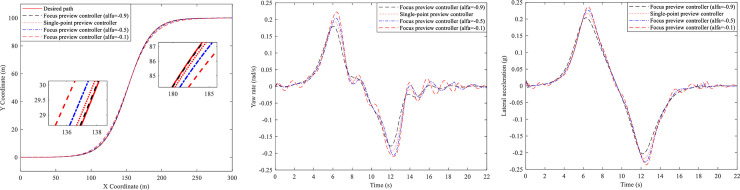
Single lane change simulation. (A) Trajectory curves. (B) Yaw rate curves. (C) Lateral acceleration curves.

In this section, the simulations of the active steering control of the tractor and the trailer at high speed (u = 20m/s) are carried out by Simulink/MATLAB. Firstly, the tractors with different controllers are simulated, and their curves of trajectory, yaw rate and lateral acceleration are presented in Fig 5.

It can be seen from Fig 5A that the tractor with focus preview controller (*α* = −0.9) can track the path most accurately, followed by the tractor with single-point preview controller, the tractor with focus preview controller (*α* = −0.5), and the tractor with focus preview controller (*α* = −0.1). And Fig 5B and 5C show that both of the peak value of the yaw rate and lateral acceleration of the tractor with focus preview controller (*α* = −0.9) are the minimum, followed by the tractor with single-point preview controller, the tractor with focus preview controller (*α* = −0.5) and the tractor with focus preview controller (*α* = −0.1).

From the above analysis, we can see that the focus preview controllers with different fractional orders have different maneuvering stability. That is to say, the tractor with focus preview controller (*α* = −0.9) can make the tractor have better characteristics than that with the single-point preview controller at high speed.

**Fig 6:**
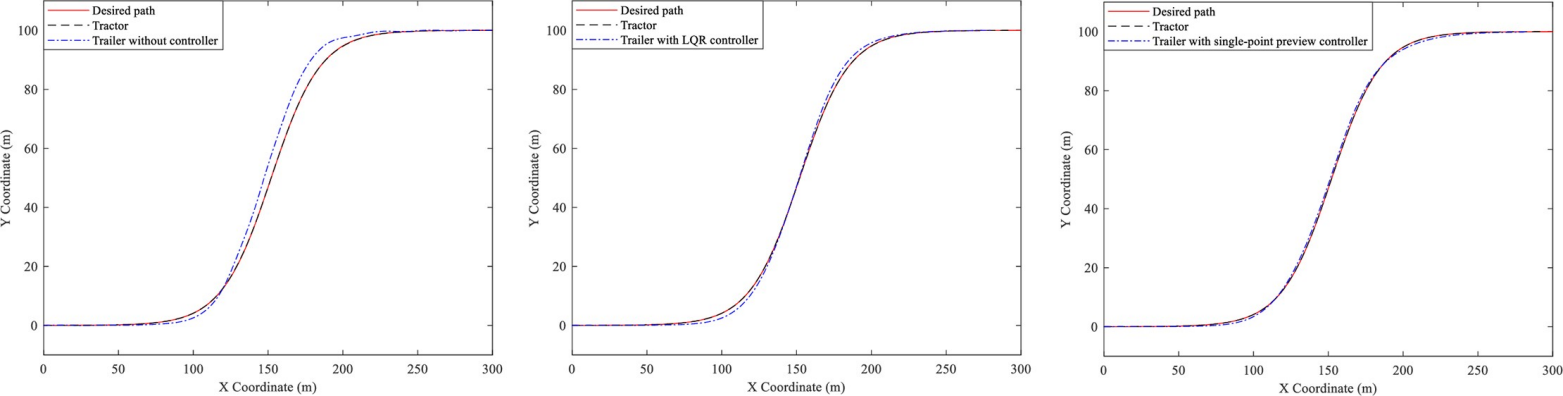
Trajectory curves. (A) Trailer without controller. (B) Trailer with LQR controller. (C) Trailer with single-point preview controller.

**Fig 7:**
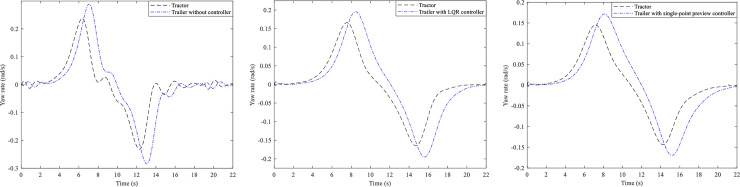
Yaw rate curves. (A) Trailer without controller. (B) Trailer with LQR controller. (C) Trailer with single-point preview controller.

**Fig 8:**
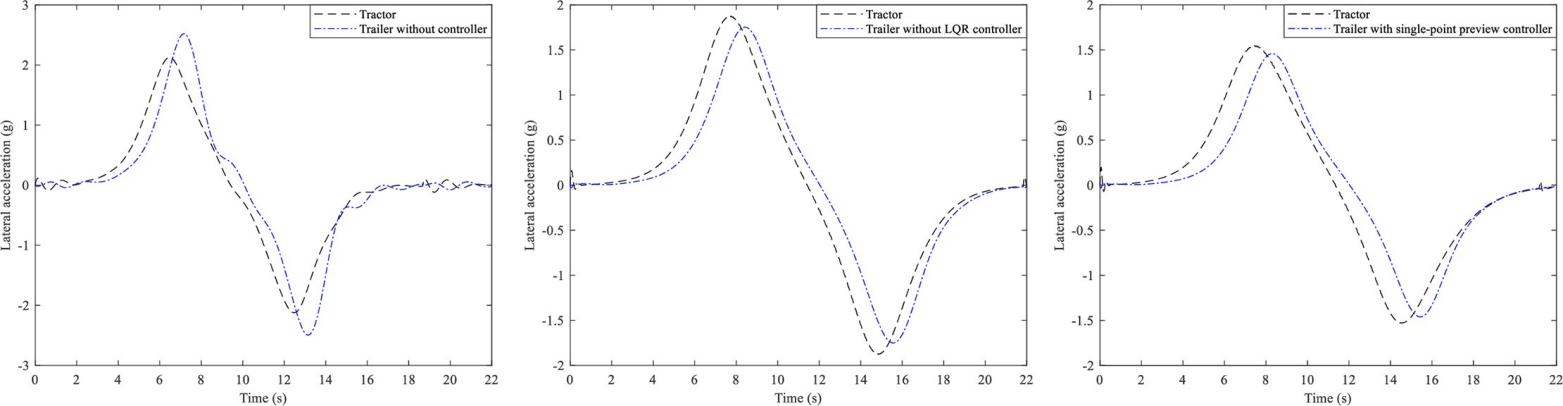
Lateral acceleration curves. (A) Trailer without controller. (B) Trailer with LQR controller. (C) Trailer with single-point preview controller.

On this basis, the active steering controls of the trailer are carried out. Here three different of AHVs are simulated, i.e., trailer without controller, trailer with LQR controller and trailer with single-point preview controller, however whose tractors all adopt the focus preview controller (*α* = −0.9). And the trajectory, yaw rate and lateral acceleration of the tractors and the trailers are presented in Figs 6–8.

From Fig 6, it can be seen that all tractors of the three AHVs can follow the desired path very well. As Shown in Fig 6A, the trajectory of the trailer without controller is very different from the desired path. And Fig 6B and 6C show that the deviation between the path of the trailers with LQR controller and the desired path is reduced, especially that the trailer with the single-point preview controller can track the desired path even better.

Fig 7 presents the yaw rates of the three kinds of AHVs and we can see that the yaw rates vary for all of the tractors. For the AHV whose trailer cannot steer, the peak values of the tractor and trailer are 0.229 and 0.28 rad/s, respectively. For the AHV with LQR controller of the trailer, the peak values of the tractor and the trailer are 0.167 and 0.195 rad/s, reduced by 27.1% and 30.4%. For the AHV with single-point preview controller of the trailer, the peak values of the tractor and the trailer are 0.145 and 0.172 rad/s, reduced by 36.7% and 38.6%.

Fig 8 illustrates the lateral accelerations of the three different AHVs. For the AHV whose trailer cannot steer, the peak values of the tractor and the trailer are 2.09 and 2.45 m/s2, respectively. For the AHV with LQR controller of the trailer, the peak values of the tractor and the trailer are 1.88 and 1.76 m/s2, reduced by 10% and 28.2%. For the AHV with single-point preview controller of the trailer, the peak values of the tractor and the trailer are 1.61 and 1.46 m/s2, reduced by 15.3% and 33.1%. According to the definition, the RWA of the three different of AHVs can be obtained as 1.17, 0.94, and 0.91.

Based on the above analysis, we can see that the single-point preview controller of the trailer has better control effect than LQR controller of the trailer.

### 6.2. 90^o^ turn maneuver

**Fig 9:**
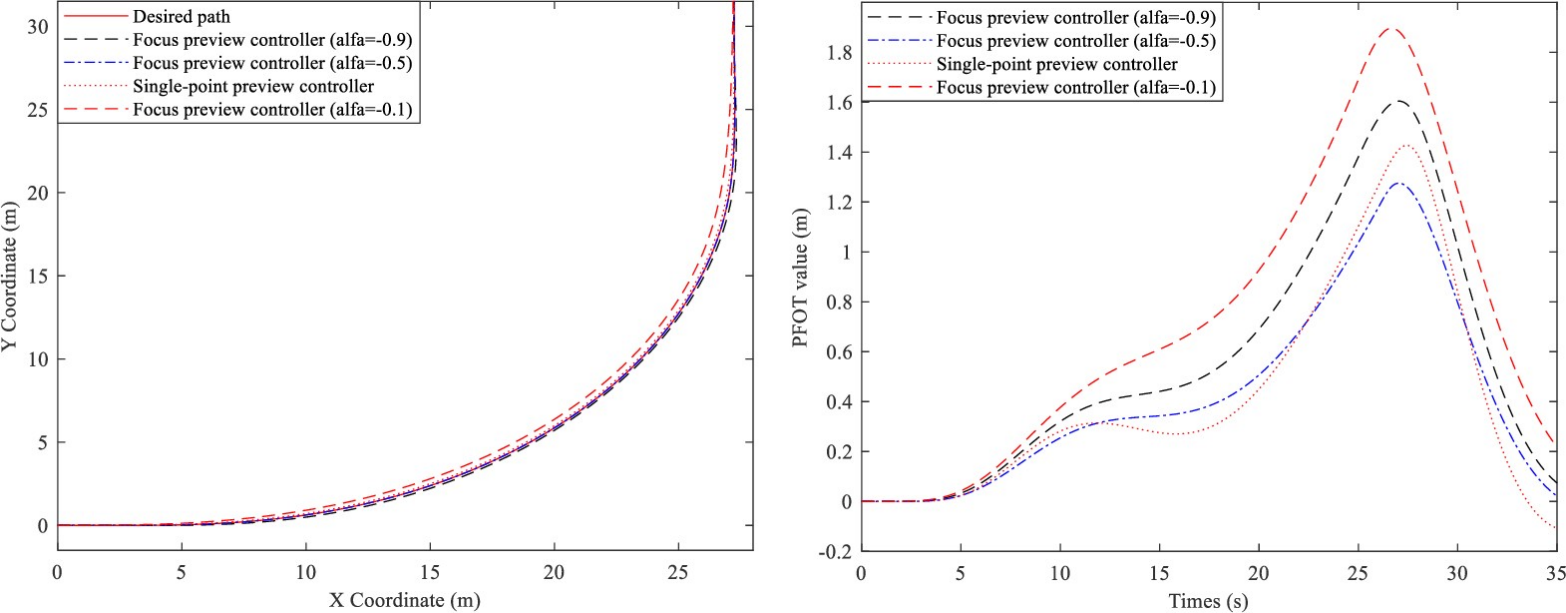
90° turn simulation. (A) Trajectory curves. (B) POFT values.

In this section, the simulations of the active steering control of the tractor and the trailer at low speed (u = 2.22m/s) are carried out by Simulink/MATLAB. Firstly, the tractors with different controllers are simulated, and Fig 9 show their trajectory and PFOT value.

From Fig 9A, the conclusion can be drawn that the tractor with focus preview controller (*α* = −0.5) can track the path very well, followed by the tractor with single-point preview controller, the tractor with focus preview controller (*α* = −0.9), and the tractor with focus preview controller (*α* = −0.1). And Fig 9B demonstrates that PFOT peak values of the tractor with focus preview controller (*α* = −0.1), tractor with focus preview controller (*α* = −0.9), tractor with single-point preview controller and tractor with focus preview controller (*α* = −0.5) are 2.03, 1.69, 1.47 and 1.34m, respectively.

In a conclusion, the focus preview controllers with different fractional order, *α*, can make the tractor have different path tracking ability. And at low speed, the tractor with focus preview controller (*α* = −0.5) can make the AHV have better characteristics than that with the single-point preview controller.

**Fig 10:**
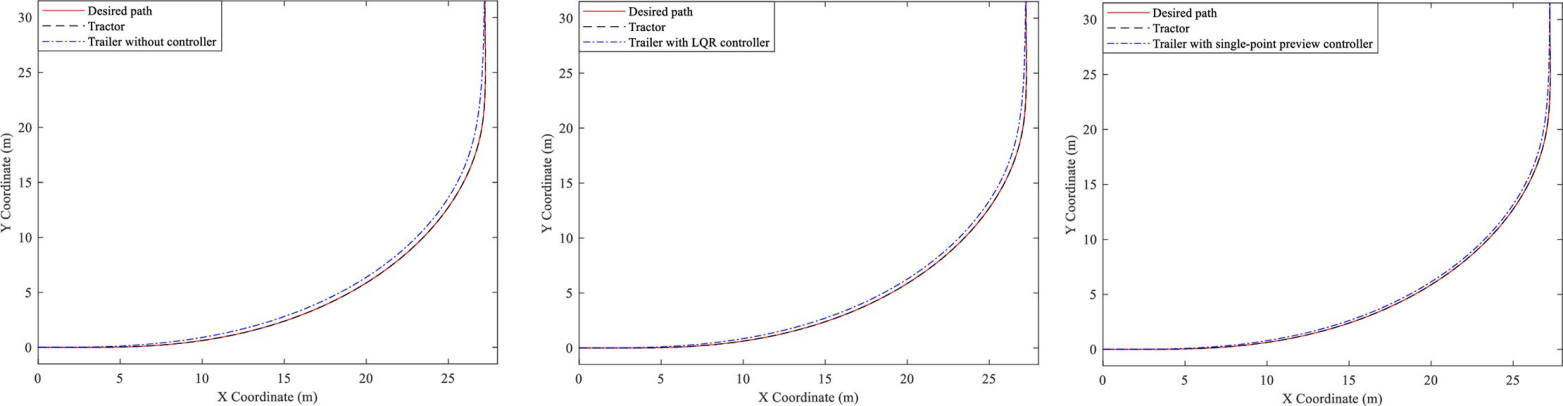
Trajectory curves. (A) Trailer without controller. (B) Trailer with LQR controller. (C) Trailer with single-point preview controller.

**Fig 11:**
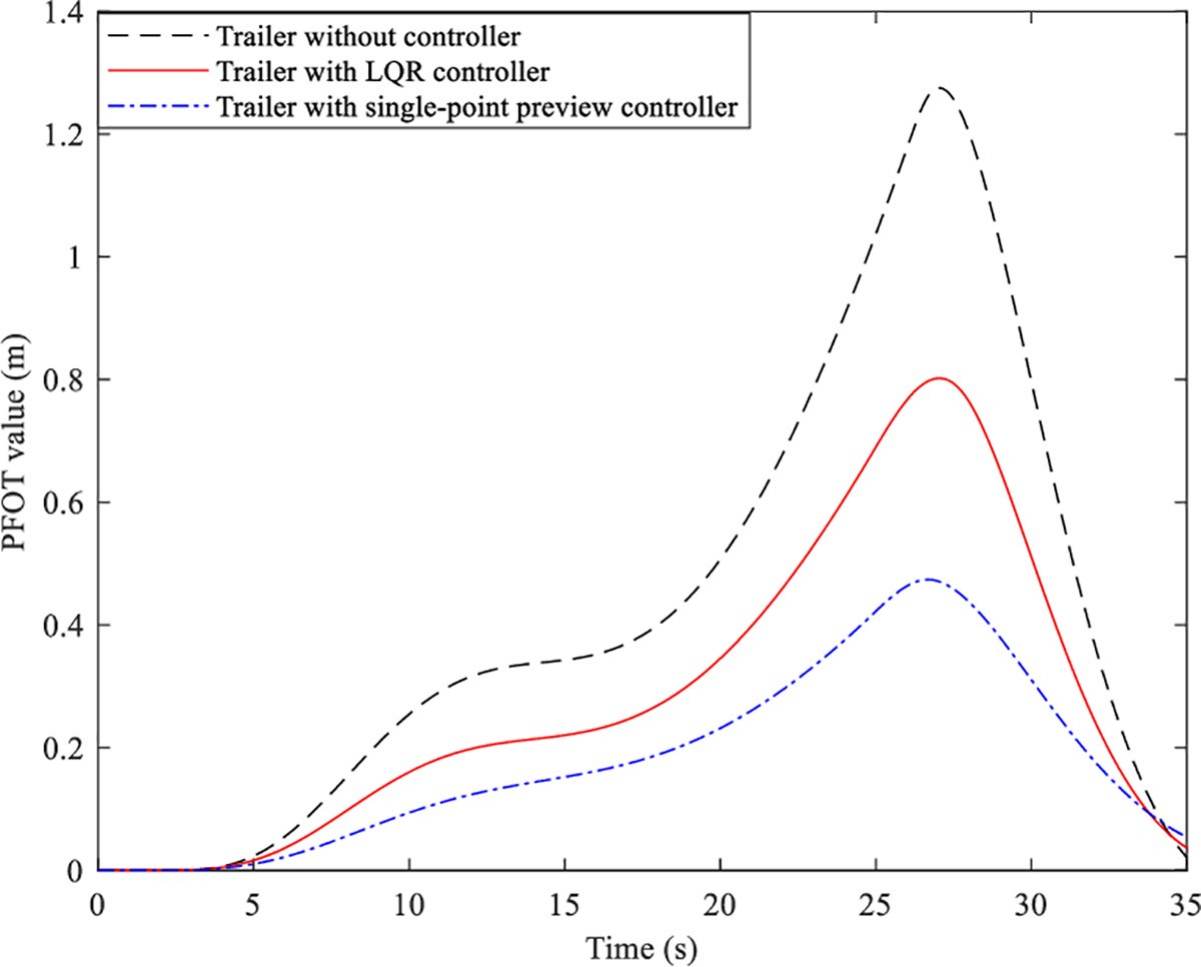
PFOT value of three kinds of AHVs.

Then three different AHVs without/with different controller of the trailer are simulated, and their trajectory and PFOT value are illustrated in Figs 10 and 11.

Form Fig 10 we can see that all of the tractors of the different AHVs can follow the desired path very well. However, the trajectory of the trailer without controller is different from the desired path. And the deviation between the path of the trailers with LQR controller and the desired path is reduced apparently, especially the deviation between the path of the trailers with single-point preview controller and the desired path. Fig 11 presents the PFOT value of the three kinds of AHVs. It can be seen that the peak value of the AHV whose trailer cannot steer is 1.34m. The peak value of the AHV with LQR controller of the trailer is 0.84m, reduced by 37.3%, and that of the AHV with single-point preview controller of the trailer is 0.51m, reduced by 61.9%. That is to say, the single-point preview controller of the trailer has better control effect than LQR controller of the trailer.

Based on the above analysis, the focus preview controller for the tractor can improve the path tracking and lateral stability of the tractor at different speeds by changing the fractional order. On this basis, the LQR control of the front wheel of the trailer can not only improve the trajectory of the trailer, but also improve the yaw rate and lateral acceleration of the AHV. In addition, the single-point preview control of the front wheel of the trailer can make the AHV have better kinematic response and lateral stability than the former.

## 7. Conclusion

The active steering control of the AHV is studied. Considering the driver’s sight characteristics, the focus preview controller for the tractor is designed to provide the steering input to make it travel along the desired path. And simulation results indicate that the focus preview controllers with different fractional order for the tractors have different lateral stability and path tracking ability. Similar to the driver’s single-point preview, a single-point preview method based on the articulation angle of the tractor and the trailer is proposed for the trailer. At high speed, the tractor with focus preview controller (*α* = −0.9) have good lateral stability. Compared with the LQR controller, the trailer with single-point preview controller has better lateral stability. At low speed, the tractor with focus preview controller (*α* = −0.5) have good path tracking ability. Compared with the LQR controller, the trailer with single-point preview controller has better path tracking ability. In the future, the roll of the AHV and the nonlinear characteristics of the tires can be taken into account.
